# Attentive Processes and Blood Lactate in the Sambo

**DOI:** 10.3390/ijerph19031113

**Published:** 2022-01-20

**Authors:** Marinella Coco, Andrea Buscemi, Matej Tušak, Vincenzo Perciavalle, Alfio Nifosì, Paolo Cavallari, Donatella Di Corrado, Valentina Perciavalle

**Affiliations:** 1 Department of Biomedical and Biotechnological Sciences, University of Catania, 95123 Catania, Italy; 2 Study Center of Italian Osteopathy and Horus Social Cooperative, 95100 Catania, Italy; andreabuscemi@virgilio.it; 3 Department of Social and Humanistic Sciences in Sport, Faculty of Sport, University of Ljubljana, 1000 Ljubljana, Slovenia; matej.tusak@fsp.uni-lj.si; 4 Department of Sport Sciences, Kore University, Cittadella Universitaria, 94100 Enna, Italy; perciava@libero.it (V.P.); donatella.dicorrado@unikore.it (D.D.C.); 5 Polisportiva City Gym, 97100 Ragusa, Italy; alfiocitygym@gmail.com; 6 Department of Pathophysiology and Transplantation, Human Physiology Section, University of Milan, 20122 Milan, Italy; paolo.cavallari@unimi.it; 7 Department of Educational Sciences, University of Catania, 95100 Catania, Italy; valentinaperciavalle@hotmail.it

**Keywords:** attentive processes, blood lactate, sport

## Abstract

Background: Sambo is a martial art and combat sport that originated in the Soviet Union. There are two main stiles, Sport Sambo and Combat Sambo which resembles modern mixed martial arts. Very little literature is available about physiological aspects of Sambo and, in particular, on the possible effects on cognitive domains. The purpose of the present research was to determine if there is a correlation between a blood lactate increase and the intensity and/or selectivity of attentions. Methods: Sixteen male athletes practicing Sambo for at least 5 years participated voluntarily in the study. Each athlete had to sustain, with an interval of one week, both a Sport Sambo match and a Combat Sambo match, each lasting 5 min. Blood lactate levels as well as attentive capacities were evaluated at three different times: at rest, i.e., 5 min before the start of the session (pre), at end of the session and 15 min after its conclusion. Reaction time protocol was used to evaluate the intensity of attention, whereas divided attention was assessed for analyzing the selectivity of attention together with errors and omissions. Results: Concerning Sport Sambo, blood lactate was 1.66 mmol/L (±0.55 SD) before the session, reached a mean value of 3.40 mmol/L (±0.45 SD) at the end of the session (end) and returned to values similar to initial ones (a mean value of 1.98 mmol/L (±0.37 SD) after 15 min (15-end). None of the attentive parameters examined, showed statistically significant differences. Conversely, for Combat Sambo, it was found a significant increase in blood lactate levels that went from 1.66 mmol/L (±0.55 SD) before the session (pre), to 4.76 mmol/L (±0.60 SD) at the end (end) and then back to values similar to those observed before the session 15 min after its conclusion (15-end), i.e., 1.97 mmol/L (±0.37 SD); however, after a Combat Sambo session increases in blood lactate were associated with significant worsening of attentional mechanisms. Conclusions: In conclusion, in all the participants, the worsening of attentional mechanisms was observed only after the Combat Sambo session in which blood lactate values exceeded 4 mmol/L. This figure, also known as the Onset of Blood Lactate Accumulation (OBLA), is commonly used to determine the anaerobic threshold.

## 1. Introduction

Sambo is a martial art as well as a combat sport that originated in the Soviet Union; the word Sambo is an acronym of Russian “Samozashchita Bez Oruzhiya” (self-defense without weapons). Sambo roughly resembles Japanese judo, but uses a greater variety of moves such as ground grappling techniques and leg locks. Its development began after World War I by the Soviet Red Army to improve hand-to-hand combat abilities, thanks to the efforts of two Soviet athletes: Viktor Spiridonov (1882–1944) and Vasili Oshchepkov (1893–1938). While Viktor Spiridonov privileged a style based on strength, Vasili Oshchepkov, who had studied judo in Japan with Jigoro Kano (1860–1938), preferred a softer style. Anatoly Kharlampiyev (1906–1972), a pupil of Vasili Oshchepkov, helped to transform Sambo from a martial art into a sporting discipline.

The International Sambo Federation (FIAS) recognizes three main styles [1], sport Sambo, combat Sambo, and beach Sambo. Sport Sambo is mainly characterized by specific throws, holds, arm and leg locks. In addition to the technique of sports Sambo in combat Sambo, rules allow blows with the hands, legs and head, as well as choking. Beach Sambo bouts are held only in the standing position.

Very little in literature is present about physiological aspects of Sambo, e.g., [2,3], in particular, on lactate production during performance and on possible effects on cognitive domains.

Although about two hundred years have passed since the studies carried out by the Swedish chemist Carl Wilhelm Scheele and the subsequent discovery of lactic acid, lactate continues to be a much-studied molecule. Lactic acid was considered for a long time a waste product of metabolism and the main cause of muscle fatigue [4]. 

However, the depletion of muscle glycogen seems to be critic for the peripheral fatigue, also considering that, fatigue-related metabolites (such as ADP, Pi, and H+) do not accumulate as exercise progresses, in spite of the fact that peripheral fatigue gradually develops [5].

Over the last few decades, it has been observed that brain cells, in particular astrocytes, produce lactate which they transfer to neurons. These latter use it as a metabolite, like glucose, in order to cope with the increase in energy needs, both in physiological conditions that it does not.

The lactate produced by the muscular system during physical exercise is released and later absorbed by muscles, heart, liver and brain, to be oxidized as a useful metabolite [4,6].

Brooks was the first to introduce the concept of lactate shuttle mechanism by which lactate, with appropriate MCT transporters, is conducted in different parts in order to be used as a metabolite [7].

It is generally recognized that after an intense exercise, blood lactate levels rise, indicating the intensity of the performance instead of muscular fatigue [4]. High levels of blood lactate, resulting from an intense exercise or an intravenous infusion of lactate solution, were found to have a negative correlation with two aspects of the attention, intensity and selectivity [8,9]. 

Why the attention? Attention is a cognitive domain that has been studied extensively in sports, as it is known that attention significantly influences sports performance [10,11,12,13].

The studies of Mackworth [14] have clarified the existence of two different types of attention: one due to the experience of the physical and social environment (regulated by the activity of the frontal lobes) and an involuntary one (regulated by the flow of external stimuli) independently of experience.

Attention can be considered as a set of processes, through which information processing and decision-making activities are controlled [15].

These processes involve specific parameters such as intensity (vigilance and sustained attention) and selectivity (broad, concentrated and divided).

Furthermore, it can be observed that general attention is characterized by the evaluation of a large amount of information, while selective attention involves the discrimination of stimuli and/or the selection of a small number of them [16].

Attention represents, for almost the multitude of sporting disciplines, a feature of considerable importance. Being ready and vigilant in order to respond to an offense, to shoot for a basket, to go off to shoot, etc. in addition to specific muscle characteristics, can make a difference [17,18,19,20,21,22,23].

The authors Furfley and Wood [24] even claim that an athlete’s attention is the key to success in sports performance.

This research’s goal was to determine if there is a correlation between a blood lactate increase and the intensity and/or selectivity of attentions. To this end, we measured both blood lactate levels and attentional abilities of participants before, at the end, and after 15 min of a Sambo session.

## 2. Materials and Methods

### 2.1. Participants

The study included sixteen volunteer male athletes who had been practicing Sambo for at least 5 years. The volunteers were fully informed about the study’s goal and signed an informed consent that was written in accordance with the ethical standards outlined in the Helsinki Declaration (revision 2013) and approved by our Institution’s Ethical Committee.

In the three days leading up to the experimental session, all participants were requested not to make any substantial changes in their habits (food intake, stimulants, physical activity, etc.). All competitors were obliged to have a certificate of suitability for competitive sports given by a Sports Medicine professional.

### 2.2. Procedure

The experimental protocol required that each athlete had to sustain, with an interval of one week, both a Sambo sport match and a Sambo combat match. Half of the participants, randomly selected, had to take first he Sambo sport match and then the Sambo combat match, while the other half had to do the opposite.

The victory can be: (a) total; (b) by superiority; (c) on points; (d) technical; (e) on warnings; (f) by the opponent’s elimination for passivity.

In the present study, both a Sport Sambo and Combat Sambo session last 5 min.

All measurements were made in three different moments: at rest, i.e., five minutes before the start of the session (pre), at the end of the session, and 15 min after it ended.

Blood lactate levels were measured using a portable analyzer called “Lactate Pro 2” (Arkray, Japan) on a drop of blood obtained by the subject’s index finger.

Two tests were used to evaluate attention, according to the Zimmermann and Fimm [25] attentional test procedure. The Attention and Concentration Tasks (ACT) [26] were used with the validated Italian version.

The ACT is composed of a set of tasks that were created specifically for evaluating different attentional abilities using a simple computer.

The main issue in this sort of experiment is that attentional tests must be completed in a short period of time (less than 4 min in total), so that the evaluation can actually occur with higher blood lactate levels. Indeed, blood lactate levels have been found to be halved 7 min after an exhausting workout [27].

The intensity of attention was measured using a reaction time (RT) methodology. The RT technique requires the participant to answer as rapidly as possible by pushing the spacebar when the symbol on the monitor target “star” appears at random intervals.

Divided attention was used to analyze attention selectivity. A dual activity was conducted, which allowed for the parallel identification of auditory and visual sort. An object would appear on the computer screen and then the name of an object would be spoken from a loudspeaker. Only if the name coming from the loudspeaker was that of the object presented on the screen the subject had to press the space-bar as fast as possible. The test allows to evaluate the role of the interferences on the divided attention through three variables: number of errors, the number of omissions and performances duration (execution time, ET).

### 2.3. Statistical Analysis

For the purpose of this study, we have chosen 16 Sambo male athletes with anthropometric characteristics very similar (see Table 1). However, such a limited number of subjects did not allow to apply the classic formula for determining the sample size. In that meaning, non-parametric statistics were preferred to parametric statistics.

Data were gathered and averaged before being contrasted with a one-way repeated measure ANOVA and the Dunn’s Multiple Comparison Test. The significance level was set at *p* = 0.05, and all data were provided as mean standard deviation (SD). All analyses were carried out using the GraphPad Prism 6:00 version for Windows (GraphPad Software, La Jolla, CA, USA, www.graphpad.com, accessed on 21 October 2021).

## 3. Results

**Figure 1:**
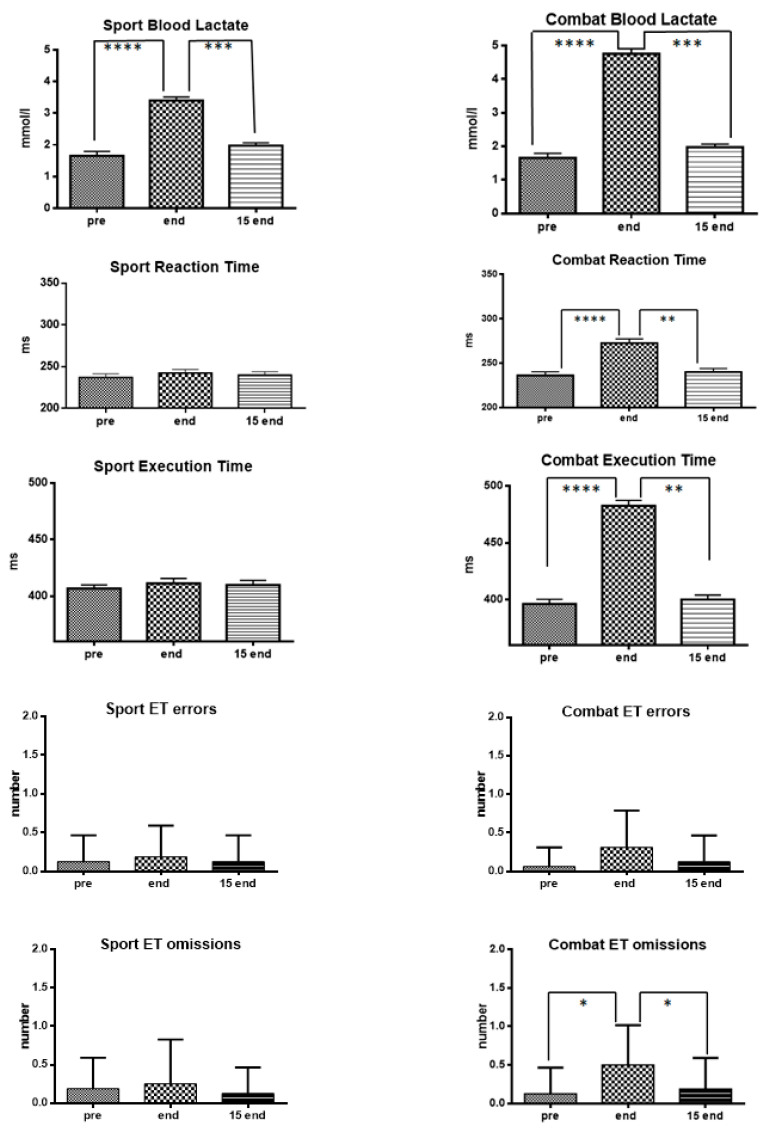
Changes of attentional processes in relation to blood lactate after a Sport Sambo session (**left**) and a Combat Sambo session (**right**). Mean values (±standard deviation) of blood lactate, reaction time, execution time, number of errors, and number of omissions measured at rest (pre), at the conclusion of the session (end) as well as 15 min after its conclusion (15 end). Symbols: * *p* < 0.05; ** *p* < 0.01; *** *p* < 0.001; **** *p*< 0.0001.

Figure 1 shows the results observed after a Sport Sambo and Combat Sambo session. As can be seen, both Sambo sessions result in a considerable increase in blood lactate levels. The observed blood lactate values in the Sport Sambo, that were 1.66 mmol/L (±0.55 SD) before the session, exhibited a significant increase at the end of the session (end), reaching a mean value of 3.40 mmol/L (±0.45 SD), and then returning to values similar to initial ones (a mean value of 1.98 mmol/L (±0.37 SD) after 15 min (15-end). It can be seen that, during the session of sport Sambo, none of the parameters examined, i.e., RT, ET errors and omissions, showed statistically significant differences.

Also for the Combat Sambo it was found that the session causes a significant increase in blood lactate levels that went from 1.66 mmol/L (±0.55 SD) before the session (pre), to 4.76 mmol/L (± 0.60 SD) at the end (end) and then back to values similar to those observed before the session 15 min after its conclusion (15-end), i.e., 1.97 mmol/L (±0.37 SD). However, in contrast to what was observed after a Sport Sambo session, after a Combat Sambo session increases in blood lactate were associated with significant worsening of attentional mechanisms, particularly RT, ET, and number of omissions.

## 4. Discussion

It is known that, during maximal exercise, at the level of the CNS, an increase in the demand for oxygen, glucose and lactate occurs; as Dalsgaard suggests, this indicates that increased brain activation is associated with the increase in physical activity [28,29].

Gonzalez-Alonso et al. observed that a marked increase in glucose, O2, and lactate utilization by the brain is associated with impaired tissue oxygenation in the frontal cortex [30].

It has been suggested that a maximal exercise induces an increase in plasma catecholamines which, in turn, influences central catecholamines neurotransmitters via the vagal/nucleus of the solitary tract pathway capable, by inducing in this way changes on cognitive domains [26,27]. However, in a previous study carried out on a subject completely immobile, it has been observed that the same changes that an exhaustive exercise produced on attentional mechanisms were also induced by intravenous infusion of lactate. This led to the conclusion that, regardless of the catecholamine theory, a rise in blood lactate is capable of worsening attentional processes on its own.

Attention, in this regard, represents that cognitive domain that can allow us to verify the incoming information [31], allowing us to focus only on the information that is relevant at that given moment, allowing us to realize the goals we had previously set and to ignore information that is irrelevant or even potentially interfering with it. The frontal lobe is the portion of our brain that plays a critical role in attentional processes.

Iidaka and collaborators observed that during the performance of physical exercise, there is a bilateral increase of cerebral blood flow in the dorsolateral prefrontal areas, region involved in selectivity rather than intensity of attention [32].

An interesting aspect of the current research was that in all the participants the worsening of attentional mechanisms was observed only after the Combat Sambo session in which blood lactate values exceeded 4 mmol/L. This value, called OBLA (Onset of Blood Lactate Accumulation), is commonly used to express the anaerobic threshold. 

Clearly, Combat Sambo has more anaerobic activities than Sport Sambo such as to justify the greater increase in lactate production during exercise, the decrease of both the intensity and selectivity of attentional capacity seen in the current study appears to confirm what has previously been observed in both maximal [33] and submaximal [34] activities.

The capacity to pay attention is an essential requirement during the development of sport performance in some cases, it turns out to be a necessary component of sporting success. It is important to note that these participants should have pretty limited attentional performances when undertaking Combat but not Sport Sambo.

The main limitations of the present study are firstly the small number of participants (*n* = 16) and, furthermore, the fact that they were all male and with very close values of age and weight.

## 5. Conclusions

In conclusion, the current study’s findings confirm that the decrease of both selectivity and intensity of attentional capacity reported in the current research appears to back up what has previously been seen in the highest level of performance, as well as submaximal exercises [34,35]. 

It can be concluded that the role played by physical exercise in reducing the blood flow of the frontal cortex, and the concomitant increase in lactate in the blood, could modify both the selectivity and the intensity of attentional mechanisms.

## Figures and Tables

**Table 1:** Sambo athletes’ anthropometric characteristics. Mean ± *s* (range).

Subject	Age (Years)	Height (cm)	Weight (kg)	BMI
mean	24.69	172.75	74.31	24.89
SD	3.07	4.16	4.32	0.90

BMI: body mass index; SD: standard deviation.
